# Vasectomy reversal outcomes in men after testosterone therapy

**DOI:** 10.1038/s41598-022-22823-8

**Published:** 2022-11-14

**Authors:** Jasper C. Bash, Jamie O. Lo, Akash A. Kapadia, Malachi Mason, Jason C. Hedges

**Affiliations:** 1 Department of Urology, Oregon Health & Science University, 3303 SW Bond Avenue CHH 10 Uro, Portland, OR 97239 USA; 2 Department of Obstetrics and Gynecology, Oregon Health & Science University, Portland, OR USA

**Keywords:** Testis, Hypogonadism

## Abstract

The prevalence of males on testosterone therapy (TT) seeking vasectomy reversal (VR) is rising. As medical therapy (MT) to recover spermatogenesis after TT has been previously described, our study’s objective is to present our institution’s management and outcomes of VR in men previously on TT. We performed a retrospective case series of vasectomy patients on TT with subsequent VR by a single microsurgeon between March, 2010 and March, 2022. 14 men undergoing VR during the study period met inclusion criteria. The median age at VR was 43 years with a median obstructive interval of 11 years. Median time from MT to VR was 5 months. Post-operative semen analysis was performed in 10 men and all demonstrated patency. 2 patients had very low sperm counts secondary to continuing TT following VR contrary to medical advice and 5 men with patency achieved pregnancy. Our study noted a high rate of vasovasostomy (VV) (96%) and sustained patency despite a 12-year median obstructive interval. Our findings support favorable outcomes with less stringent VV indications after MT in patients previously on TT that desire VR. The use of MT reduces the recommended wait times for VR after TT discontinuation by more than half.

## Introduction

Nearly half a million men of reproductive age undergo vasectomies annually in the United States, at an average age of 31 years old. Of these, approximately 6–10% of men express decisional regret and desire a vasectomy reversal (VR) within 10 years^1^. Management and counseling of these patients desiring VR has become progressively more difficult as many are on testosterone therapy (TT). It is well known that exogenous testosterone has contraceptive affects due to its potent inhibitory effects on the hypothalamic–pituitary–gonadal (HPG) axis, resulting in gonadotropin suppression^2^. This gonadal suppression leads to atrophy of the testicular germinal epithelium and oligo- or azoospermia^2^. It is also well established that the suppressive effects of TT are dose, route, and duration dependent^3^. After discontinuation of TT, two-thirds of men will experience return to sperm concentrations of > 20 million/mL within 6 months, while 10% require 1–2 years, and some never have spontaneous return of fertility^2^.

The diagnosis of male hypogonadism and its treatment with TT has become increasingly common. Idiopathic hypogonadism affects a third of men between 45 and 54 years of age^4^ and the prevalence of TT use for treatment has increased, on average 15.5% annually, in the last two decades. In men 18–45 years old there has been a four-fold increase in TT compared to older men in which there has only been a three-fold increase^5^. Despite increasing use of testosterone replacement therapy in men of reproductive age, a clear indication for use is often absent. Previous studies^6^ have reported that only 4% of men receiving TT for presumed hypogonadism have completed the two pre-TT serum testosterone level assessments recommended by the American Urological Association and Endocrine Society guidelines. Indications reported for starting TT were not standardized and included hypogonadism (48%), fatigue (18%), erectile dysfunction (15%), depression (4%) and psychosexual dysfunction (1%)^6^.

Despite the prevalence of VRs and rising incidence of TT, there are currently no guidelines regarding the management of vasectomized patients on TT who present for VR. Medical salvage therapy with clomiphene citrate (CC) or human chorionic gonadotropin (hCG) have been utilized to increase intratesticular testosterone and foster spermatogenesis, but it is a challenge to monitor the effectiveness given that the post-VR anatomy precludes clinically useful semen analysis*.* Monitoring gonadotropin levels is also of low utility because they do not correlate with spermatogenesis while on TT^7^. Thus, the optimal timing of VR following cessation of TT is unknown, with the existing literature recommending waiting 6 months–1 year.

To our knowledge, this is the largest case series to date to review the management and post-operative outcomes of VR and pregnancy outcomes after medical therapy in men previously on TT at our institution. As such, the findings of our study will add to the paucity of literature and provide an algorithm to aid providers in evaluating and counseling these complex patients.

## Methods

We conducted a retrospective study of consecutive VR cases performed in a single, large academic urology practice. Our study’s protocol (#00010007) was approved by the OHSU Intitutional Review Board. Because the research is no more than minimal risk to the subjects and does not adversely affect the rights and welfare of the subjects, the need for informed patient consent was waived by the OHSU Institutional Review Board. All experiments were performed in accordance with relevant guidelines and regulations. All microsurgical VRs were performed by a single fellowship-trained microsurgeon (J.C.H) from March 1, 2010 through March 31, 2022. Patients were included in the study if they had a history of TT between time of vasectomy and VR and had complete operative findings documented.

Preoperative data, including patient age at time of VR and length of obstructive interval, were abstracted (Table 1). Details of TT including dose and duration were not available for all patients, as TT was initiated by their referring outside providers prior to VR evaluation at our institution and patient self-report was not reliable in part due to length of time since stopping TT. Thus, this was not included in the analysis. After VR evaluation, patients were discontinued on TT and for patients with T levels less than 300 ng/dL, medical therapy with CC 25–50 mg daily and hCG 1000–2000 units administered subcutaneously twice weekly was recommended. Although both CC and hCG were recommended to all patients with T levels less than 300 ng/dL, some underwent dual treatment, some elected for CC only, and others continued TT without further medication support. Testosterone was checked 4 weeks following initiation of medical therapy for dose adjustment goal T level 300–800 ng/dL. The decision to move forward with VR was determined by clinical exam and/or post-medical therapy assessed 3 months. The regimen and the length of time on medical therapy were recorded.

**Table 1:** Patient characteristics and outcomes.

Patient	Obstruction interval (years)	Choice of MT	Time on MT (months)	Vasal fluid (L, R)	Micro sperm (L, R)	Type of reversal (L, R)	Patency	Pregnancy
1	14	CC	7	Clear, clear	+/+	VV, VV	Yes	Yes
2	7	CC	11	Clear, clear	+/+	VV, VV	Yes	Yes
3	18	None	N/A	Creamy, creamy	+/+	VV, VV	Yes	No**
4	11	CC	5	Opalescent, pasty	+/−	VV, EV	Yes	Yes*
5	7	CC	5	Clear, clear	+/+	VV, VV	Yes	Yes
6	30	CC, hCG	5	Opalescent, opalescent	+/+	VV, VV	Yes	Yes
7	12	CC, hCG	4	Clear, clear	−/−	VV, VV	No	No
8	13	CC	2	Opalescent, opalescent	+/+	VV, VV	Yes	No
9	10	CC	3	Opalescent, opalescent	+/+	VV, VV	Yes	No
10	10	CC	1	Opalescent, opalescent	+/+	VV, VV	Yes	N/A
11	7	CC, hCG	4	Opalescent, opalescent	−/−	VV, VV	Yes	No
12	11	hCG	4	Opalescent, clear	+/+	VV, VV	Yes	N/A
13	8	CC, hCG	6	Opalescent, opalescent	+/+	VV, VV	N/A	No**
14	15	CC, hCG	4	Opalescent, opalescent	+/+	VV, VV	Yesf	No**

*CC* Clomiphene Citrate, *hCG* Human Chorionic Gonadotropin.

Median obstruction interval is 11 years and median time on medical therapy is 5 months.

Patency is defined as presence of sperm on semen analysis.

Type of Reversal is 96% Vasovasostomy.

*IVF pregnancy resulting in spontaneous miscarriage.

**Patient has not tried to conceive.

All patients underwent microsurgical VR with either a 2-layer vasovasostomy (VV) or end-to-side epididymovasostomy (EV) based on intraoperative findings^8^. Intraoperative data including the characteristics of the intravasal fluid (gross appearance, presence or absence of sperm or sperm parts) and the methods of reconstruction (VV vs. EV) was obtained. Semen analysis was obtained 4 to 6 months after VR unless pregnancy was already achieved. Phone interviews were conducted in January, 2022 to assess the pregnancy status of couples postoperatively.

### Conference presentation

Presented orally at the American Urological Association Annual Meeting, New Orleans, Louisiana. May 14, 2022.

## Results

### Patient characteristics

Of all men who underwent VR during the study period, 14 met our study’s inclusion criteria. Median age at VR was 43 years old (IQR 37.8–49) with a median obstructive interval of 11 years (IQR 7.4–14). Prior paternity had been achieved in 13 (93%) men. Median partner age at time of VR consultation was 32 years old (IQR 28.3–36), and median prior TT duration was 24 months (IQR 12–60). The median duration of medical therapy was 5 months (IQR 3.8–6.3) prior to VR and included CC alone in 7 (50%) men, CC with hCG in 5 (36%) men, and no medical therapy in 1 (7%) man.

### Operative findings

Of the 28 vasa deferens repaired, sperm was noted intraoperatively in 21 (75%) of them. Gross characteristics of the vasal fluid were as follows, in descending order of favorability for pregnancy: clear 32%, opalescent 57%, creamy 7%, and pasty 4%. There was an 86% concordance rate between vasa deferens of the same man. VV was performed in 27 (96%), with VE for the remaining 1 (4%).

### Patency

Patency was defined by the presence of sperm on semen analysis after VR. Of the 14 patients who underwent VR, 2 achieved pregnancy prior to their post-operative semen analysis, so patency was assumed. One patient is hypogonadal on hCG treatment, thus his post-operative semen analysis has been deferred. Of the remaining 11 patients, 8 had post-operative semen analyses within normal limits. Of the 3 patients without normal semen analyses, two resumed their TT postoperatively: 1 had severe oligospermia (< 5 million/mL), and the other had sperm fragments on semen analysis. The third has yet to complete his first post-operative semen analysis.

### Pregnancy

Five men successfully achieved live births, all of whom had post-operative semen analysis results consistent with patency (Table 1). One couple also had female factor infertility and underwent in-vitro fertilization, but the pregnancy resulted in miscarriage. Another couple decided not to pursue pregnancy. One patient remained hypogonadal on hCG treatment, two patients resumed TT, one patient was lost to follow up, and three patients underwent VR recently within the last three months.

## Discussion

Our study found a very high rate of successful VV in men undergoing VR after a short interval of medical therapy after TT, in spite of a long obstructive interval and non-favorable intraoperative findings. This suggests that TT is protective to the vas, and intraoperative decision-making should be modified in this population.

The increase in TT among young men will lead to increasing consultations for VR in the setting of prolonged TT. Despite a rise in use, there is a paucity of guidelines or best practice statements to guide the timing of medical and surgical therapies. TT has increased 15% annually^6^: fourfold in men 18–45 years old compared to threefold in older men^5^. The prevalence of both vasectomy and vasectomy regret is also high in this group, as 15.9% of American men aged 36–45 years have undergone a vasectomy and 19.6% of vasectomized men in the US desire future children^9^.

The gross and microscopic intraoperative findings of vasal fluid at the time of VR have been well-described as predictors of reversal success and used to guide surgical decision-making regarding VV or VE^10^. The gross characteristics, by order of decreasing pregnancy success, are opalescent, creamy, clear, then pasty. In the setting of microscopic identification of motile sperm, intact non-motile sperm, and sperm with short tails, a VV is often performed^11^. However, in the absence of sperm or the presence of only sperm heads, an EV is completed. The intraoperative findings of our patients on TT did not correlate with their postoperative semen analysis as we would have expected based on the above. Two patients with early pregnancy had clear/clear fluids, which we would usually consider a disadvantageous finding. The other six patients who completed post-operative semen analysis and did not resume TT had excellent semen analysis results with a wide range of intraoperative findings: 7 opalescent, 2 clear, 2 creamy, 1 pasty. The only patient to undergo a VE for pasty fluid had a contralateral VV for opalescent fluid and had an excellent semen analysis.

The discordance between intraoperative findings and post-operative semen analysis suggests that intraoperative findings following TT should be interpreted differently, with a much higher threshold to pursue EV. TT decreases spermatogenesis, which may impact the male reproductive tract proximal to the vasal ligation (e.g. vas deferens, epididymis, and rete testis). As a result, these tissues may be less chronically dilated, promoting more optimal healing after VR. The protective effect of TT would then be to place the ligated system in a quiescent state, until it is awakened by medical therapy and VR. Routes of TT administration with higher spikes in serum testosterone, such as intramuscular injection (IM), dampen spermatogenesis faster than those with more physiologic levels like pellet implants^12^. While IM TT decreases sperm counts in only one month, pellets lead to azoospermia in 2–4 months, suggesting that any form of long-term TT will likely provide vasal protection.

One recent retrospective series focused on 6 men undergoing VR with a history of TT^13^. Coward et al. reported a total of 3 VE and 9 VV, with 83% patency and 50% pregnancy^13^. The medical therapy used in this study was similar, although their workup included Testicular Sperm Aspiration (TESA) for two patients with “uncertain recovery of spermatogenesis based on physical examination and hormone response.” Notably their only patient without a patent semen analysis also underwent bilateral VE.

Coward et al. recommend an algorithm to manage spermatogenesis salvage therapy when treating men who desire VR after TT that includes discontinuation of TT and initiation of CC with or without hCG for 3 months, after which hormone evaluation (testosterone, luteinizing hormone, follicle stimulating hormone) and physical exam should be performed^13^. Based on our results and the literature described above, we support the algorithm described by Coward et al., and propose a narrower indication for TESA, which should be performed if serum testosterone is persistently low or serum testosterone is appropriate but patients still feel symptoms of low testosterone. Serum levels of 17-hydroxyprogesterone could also be used to evaluate intratesticular testosterone levels in that subgroup^14^. Serum FSH levels could also be employed at the initial evaluation to assess the current degree of spermatogenesis suppression, with normal levels suggestive of a balanced HPG axis. While this would further shorten the time to reversal, the risk of persistent occult TT-induced azoospermia in the early stages of recovery with normal FSH might alter intraoperative findings and decisions. Thus, 3 months of empiric spermatogenesis-promoting therapy balances optimizing time to conception with surgical outcomes. The decision between VV and VE based on intraoperative findings should favor VV in these men and bilateral VE be avoided as gross vasal fluid findings are not as reliable in this population. Given that two of our patients resumed TT after VR, this suggests the need for further education at every visit regarding the adverse impact of TT on spermatogenesis.

Our findings support favorable outcomes with more liberal VV indications after medical therapy in patients previously on TT that desire VR. The use of medical therapy reduced the recommended wait times for VR after TT discontinuation alone by more than half, and the high rate of VV (96%) and sustained patency despite an 11-year median obstructive interval indicates that VR in this population is very likely to succeed.

## Data Availability

The datasets generated and/or analyzed in this study are not publicly available due to patient privacy concerns, but are available from the corresponding author on reasonable request.
